# Factors affecting staff morale on inpatient mental health wards in England: a qualitative investigation

**DOI:** 10.1186/1471-244X-11-68

**Published:** 2011-04-21

**Authors:** Jonathan Totman, Gillian Lewando Hundt, Elizabeth Wearn, Moli Paul, Sonia Johnson

**Affiliations:** 1 Research Department of Mental Health Sciences, University College London, Gower Street, London, WC1E 6BT, UK; 2 School of Health and Social Studies, Institute of Health, University of Warwick, Coventry, CV4 7AL, UK; 3 The University of Warwick, Warwick Medical School, Gibbet Hill Campus, Coventry, CV4 7AL, UK; 4 Camden and Islington NHS Foundation Trust, London, UK

## Abstract

**Background:**

Good morale among staff on inpatient psychiatric wards is an important requirement for the maintenance of strong therapeutic alliances and positive patient experiences, and for the successful implementation of initiatives to improve care. More understanding is needed of mechanisms underlying good and poor morale.

**Method:**

We conducted individual and group interviews with staff of a full range of disciplines and levels of seniority on seven NHS in-patient wards of varying types in England.

**Results:**

Inpatient staff feel sustained in their potentially stressful roles by mutual loyalty and trust within cohesive ward teams. Clear roles, supportive ward managers and well designed organisational procedures and structures maintain good morale. Perceived threats to good morale include staffing levels that are insufficient for staff to feel safe and able to spend time with patients, the high risk of violence, and lack of voice in the wider organisation.

**Conclusions:**

Increasing employee voice, designing jobs so as to maximise autonomy within clear and well-structured operational protocols, promoting greater staff-patient contact and improving responses to violence may contribute more to inpatient staff morale than formal support mechanisms.

## Background

Psychiatric inpatient wards are potentially highly stressful places to work. In England, the shift towards community-based care in the post-deinstitutionalisation era has raised the threshold for admission, with more patients detained under section and shorter lengths of stay [1]. Policy makers, managers, clinicians and service users have all expressed concerns regarding the quality of inpatient care [2-4]. National audits report high rates of violence on psychiatric wards [5] and difficulties identified in a national review of acute wards [6] included high staff vacancy and sickness rates, lack of leadership from consultant psychiatrists, poor communication with community teams and limited availability of psychological treatments.

Staff morale in the NHS is important in several respects. Firstly, the NHS is one of the world's largest employers, and achieving the status of an exemplary employer has recently been defined as an important goal [7]. Secondly, the cost to the nation of the current high rates of staff sickness in the NHS is around £1.7 billion per year. Thirdly, substantial correlations have been found in healthcare settings between staff well-being and patient outcomes [7]. In inpatient mental health, there is increasing evidence that therapeutic relationships are key determinants of patient experiences [8]: staff attitudes and well-being are likely to influence these. Finally, the problems identified in UK inpatient mental health care have resulted in a series of initiatives aimed at service improvement. A growing body of 'implementation sciences' literature [9] indicates that negative professional attitudes to work are a major block to the successful dissemination of innovations intended to improve patient experiences and outcomes.

Until recently, there has been little comprehensive research on the morale of NHS inpatient mental health staff, with most studies employing small samples and confined to single sites or including only mental health nurses [10-12]. The qualitative study described in this paper was the second component in a mixed methods national investigation of inpatient staff morale. The first part of this investigation was a quantitative questionnaire survey on 100 wards across the country, reported on by Johnson and colleagues [13,14]. Findings from this quantitative study were that most NHS inpatient staff were fairly satisfied with their work and reported a sense of achievement from it. However, a substantial proportion were 'burnt out' on the 'emotional exhaustion' subscale of the Maslach Burnout Inventory [15], ranging from 29% on rehabilitation wards to 49% on acute wards.

An understanding of the factors underlying good or poor morale on wards is likely to be required for effective strategies to improve morale to be designed, but empirical examinations of these are even rarer than studies of levels of morale [11]. The quantitative study which preceded the current study and included the wards on which the current study was conducted [13,14] examined associations between indicators of morale and a range of candidate influences. The demand-control-support model [16], which proposes that work strain results from a combination of high job demands, low autonomy in the way these can be met, and low support from managers and colleagues, was largely upheld [14]. Other organisational variables which were associated with morale indicators were staff ratings of role clarity and team communication, and perceived fairness in the work environment. Experiences of bullying and violence were also highly associated with morale. Ward type and various demographic indicators were also associated with morale, but staffing levels and specific physical characteristics of the ward were not.

Quantitative data of this type illuminate potential underlying mechanisms for good and poor morale to only a limited extent. Qualitative accounts have a major complementary role in allowing an understanding of how staff make sense of their experiences at work, their views about how to improve their experiences, and the mechanisms that might underlie their responses to particular sources of stress and satisfaction. A systematic review on staff morale in 2004 showed that 38 out of 39 qualitative studies included in the review were single site case studies [10]. A qualitative study in three sites in London reported that ward staff complained of lack of autonomy and opportunities to develop an independent therapeutic role with patients. Informal peer support was the most frequently cited source of support [17,18].

The current study reports findings from a substantial multicentre qualitative investigation of inpatient staff views regarding the factors that influence their morale. Aims were to extend current understanding of the mechanisms underlying good and poor morale on inpatient wards, and to generate potential strategies for improving morale. This qualitative study was nested within a national multi-site quantitative study [13,14]: a secondary aim of the current qualitative investigation was to aid interpretation of the quantitative study's findings, and the discussion includes an examination of areas of congruence between the findings of the two studies.

## Method

This study reported here is the qualitative component of the multiple methods National Inpatient Staff Morale study, commissioned by the National Institute of Health Research Service Delivery and Organisation programme [13,14]. Multicentre ethics approval was obtained from the Hertfordshire Local Research Ethics Committee.

### Setting

Seven wards in London and the Midlands were included in this qualitative investigation. They were a purposively selected sub-sample from the 100 wards participating in the quantitative questionnaire survey. The quantitative survey involved administering a questionnaire including measures of levels of morale to staff on 100 wards in 4 English regions, selected to represent all the main inpatient mental health sub-specialties and areas with a wide range of geographical and demographic characteristics. For practical reasons related to researcher location, the seven wards participating in the current qualitative study were selected from 6 of the 18 Trusts participating in the initial quantitative study. Purposive selection within these six Trusts was based on stratification of the sample by mean morale scores obtained in the initial quantitative surveys. For each ward participating in the quantitative study, we calculated a standardised mean morale score based on all the questionnaire measures of morale^1 ^[13]. We used this to identify wards within the Trusts participating in the qualitative study that had mean morale scores in the top quartile or the bottom quartile of the 100 wards participating in the national study. Further selection among the candidate wards identified in this way was guided by the aims of including wards from a representative range of specialties and from several different Trusts. Where more than one ward was equally suitable for inclusion based on these principles, we selected the ward with the most extreme morale score. Following these principles resulted in a sample of four wards from the top quartile for morale in the national survey and three wards from the bottom quartile. Three were general acute wards admitting adults of working age in mental health crises, one a rehabilitation ward, one a forensic rehabilitation ward, one a child and adolescent unit and one a psychiatric intensive care unit (PICU).

### Sample

On six of the seven wards, we conducted two focus groups with staff. One consisted of junior staff from a range of professional backgrounds, including ward nurses, junior doctors, nursing assistants and other staff without professional qualifications, and basic grade occupational therapists (OTs). The other consisted of senior staff who worked on the ward and also had some managerial responsibility for other staff on the ward, including the ward manager and deputy ward managers, consultant psychiatrists with responsibility for beds on the ward, and, where relevant, senior members of other professions such as consultant clinical psychologists and Head OTs. In one ward focus groups were not possible due to staffing constraints so extra individual interviews were carried out.

On each ward, we also conducted individual interviews with members of staff of different seniority and professional backgrounds, and one interview with a more senior service manager not based on the ward, such as a lead nurse for a whole hospital or a service manager responsible for a group of wards. In the individual interviews, we sampled purposively to obtain the perspectives of staff from a full range of levels of seniority and professional backgrounds. The final data set consisted of 12 focus groups, 24 ward staff interviews (8 managerial staff, 16 non-managerial staff), and 7 senior manager interviews.

### Procedure

Interviews followed a semi-structured format and were conducted by trained research workers supervised by GH, SJ and MP, using a topic guide that explored positive and negative aspects of work, perceptions of staff morale on the study ward, the factors affecting staff morale, and ideas for how morale might be maintained or enhanced. The main questions were very broad and open-ended, enquiring how staff felt about their jobs, what main factors they felt influenced their feelings about work, and how their working environment might be improved. A list of prompts was used to explore views about areas not spontaneously touched on - these were identified from two sources: (a) areas identified from the literature as potentially linked to morale; and (b) expert views from the large multidisciplinary steering group for the National Inpatient Staff Morale Study [13]. They were modified following pilot application on two wards. Focus groups followed a similar format, with discussion focused on factors affecting *team*, rather than individual, morale. The main questions for discussion among group participants were their views about which are the main positive and negative influences on team morale. All staff provided written informed consent prior to participating.

### Analysis

Interviews were recorded and transcribed verbatim. Data were analysed using thematic analysis [19,20] within NVivo7 software. Analysis sought to answer initial research questions and explore emergent themes, investigating both commonalities and variations within the data. To enhance validity, a collaborative approach was adopted. A template of lower order descriptive categories was agreed on by members of the research team (SJ, GH, MP and JT) through reading the same interviews independently and discussing categories. JT coded using the template and elaborated it, with regular consultation with the whole team, into a hierarchical thematic framework

## Results

The characteristics of the participants in individual interviews are shown in Table 1 and those of focus group participants in Table 2. In total, 71 staff participated, representing a full range of mental health professions and levels of seniority, with the senior focus group and individual interview participants having worked in mental health services for a median 12 years and the junior staff focus group participants for a median 6.5 years.

**Table 1:** Characteristics of participants in individual interviews

Characteristic	Frequency
**Job**	
Ward nurse	6
Healthcare or nursing assistant	6
Charge nurse or deputy ward manager	4
Ward manager	3
Occupational Therapist (OT)	2
OT assistant, technical instructor or activity worker	2
Consultant psychiatrist	1
Modern matron (lead nurse role for a hospital)	2
Clinical director (medical manager for a group of services)	1
Senior manager of a group of services	4
**Gender**	
Male	8
Female	21
**Age group**	
Under 26	2
26-35 years	8
36-45 years	11
46-55 years	7
Over 55 years	1
**Ethnic group**	
White British	13
White Irish	2
White Other	2
Black African	4
Black Caribbean	3
Black Other	2
Asian Other	2
**Length of service on ward in months**	
Mean (standard deviation)	53 (76)
Median (range)	34 (1-312)
**Length of service in mental health care in years**	
Mean (standard deviation)	14.5 (9.6)
Median (range)	12 (1-36)

**Table 2:** Characteristics of participants in focus groups

Characteristic	Frequency
**Job**	
Ward nurse	12
Healthcare or nursing assistant	7
Charge nurse or deputy ward manager	4
Ward manager	4
Occupational Therapist (OT)	2
OT assistant, technical instructor or activity worker	2
Consultant psychiatrist	2
Non-consultant grade psychiatrist	2
Clinical psychologist	3
Child psychotherapist	1
Social worker	2
**Length of service on ward in months**	
Median (range) for senior staff group participants	48 months (1-300)
Median (range) for junior staff group participants	24 months (2-192)
**Length of service in mental health care in years**	
Median (range) for senior staff group participants	12 years (5-30)
Median (range) for junior staff group participants	6.5 years (1-23)

Identified themes relevant to staff morale and well-being were in four main categories: (a) the staff team; (b) the management and leadership context: (c) organisational structures and (d) being with patients. Below we describe themes within each of these categories. A fifth area, physical environment, will be briefly summarised here and more extensively described in a separate publication.

### (a) The staff team

Ward staff recurrently identified the composition of the front-line ward team and relationships within it as crucial for morale.

#### Staffing Levels

Staffing levels were viewed as central to morale by staff on all wards, some describing them as intermittently and others as constantly very problematic:

*We need more staff desperately and yes, that's probably the one thing more than anything else really because that would free up everything. That would free up the off-duty and the annual leave, the morale, the pressure and people would enjoy their job more*. (Nursing Assistant, PICU)

Many front-line staff felt overworked, describing the physical and emotional toll of a busy shift. Staffing levels could make it difficult to find time for a break, and to organise supervision and training, particularly on acute wards, where the risk of incidents intensified the need for adequate staff presence:

*Just getting on with the day to day work means that some of the things that might actually be more supportive for people, like meeting together...get pushed to one side*. (Consultant Psychiatrist, Acute)

A further concern on four wards was sickness absence and problems with recruitment leading to a perceived over-reliance on "bank" staff. Participants spoke of their uncertainty about the skills of bank staff, particularly regarding "control and restraint" procedures and adherence to ward routines and protocols. Staff also noticed that patients were generally reluctant to approach staff they did not know:

*I suppose the anxiety is if it kicks off, they're not going to know the best way to respond*. (OT, Child & Adolescent)

#### Peer relations and teamwork

Effective team working and good relationships with colleagues were the most highly valued positive influences on morale. Staff on two 'high morale' wards - the Rehabilitation ward and the Child & Adolescent Unit - were especially positive about a sense of shared responsibility and their reliance on peer support:

*It's probably one of the most important things that gets me out of bed in the mornings to come here, that, generally speaking, I have pretty good relationships with people here*. (Consultant Psychiatrist, Child & Adolescent)

*Oh the team here are excellent. You couldn't wish for better people and everybody gets on well and there's a mixture of sort of staff and the ideas that everybody has, so we get on ever so well, yes definitely*. (Staff nurse, Rehabilitation)

A culture of openness and acceptance, where staff are encouraged to give their views regardless of seniority, was associated with good morale:

*Sometimes nursing assistants aren't seen as part of...domestics are not seen as part of the team. Consultants are put on a pedestal and that doesn't really happen here does it? Everyone seems to have an equal opinion and an equal say*. (Student, Rehabilitation)

But some tensions were also reported from such tight-knit ward communities, where very close relationships created the risk of fallouts and cliques.

### (b) The management and leadership context

Themes emerged relating both to clinical leadership within the ward, generally perceived as originating from the ward manager and to some extent the lead psychiatrist or psychiatrists, and to the senior management team beyond the ward, such as those responsible for the hospital or mental health Trust as a whole.

#### Leadership within the ward

Senior staff stressed the importance of strong and effective leadership. Consistency in leadership, aided by effective communication within the managerial team, was thought to be reassuring for staff, whilst weak leadership was linked to ambiguity and uncertainty. On one ward, multiple references were made to the impact of a new consultant psychiatrist:

*This guy is very direct, very clear about what plans he wants in place, and he's very open and warm, and, you know, very good, so I think it's made a big difference*. (Lead nurse, Acute)

Several others on different wards made reference to the way inspiring individuals could boost morale, and the importance of a reliable leadership team:

*I think that the things that influence morale in a positive way are stability of the staff team, particularly in leadership functions... this kind of work brings its troubles but overall there's a leadership team which I think is very responsive, containing and supportive of the wider staff team and very good at its job... I think that's absolutely 90% of the whole thing*. (Clinical Director, Child and Adolescent)

#### Support and supervision

This was the most discussed issue among lead/managerial staff working on the ward. Managers and senior managers unanimously believed that formal support mechanisms and supervision are vital for a successful team. Formal supervision was said to help solidify roles and responsibilities and improve confidence. Four wards had staff support groups, on which views were mixed. Senior staff regarded them as a source of mutual emotional support but several front-line staff members said they found them uncomfortable.

Front-line staff spoke more about the value of informal support from managers than about supervision. They appreciated the visible presence of leading staff on the "shop floor", their availability for guidance and reassurance, and their responsiveness to work-related problems. On all wards there was discussion of the importance of feeling valued, with frequent comments that praise and recognition could be more forthcoming.

Support following violent incidents was seen as important by staff on every ward, not just for immediate reassurance but because it sent a message that staff were being looked after. One group talked about how they used to receive letters following an incident, which had since ceased. Although it was "the exact same letter" every time, said one person, "at least you felt they were thinking of you".

*They used to come down afterwards and check if everyone was alright and that's important, you know? The small things make a big difference*. (Staff Nurse, PICU)

The availability of formal supervision and the extent to which staff felt supported in their roles varied between wards and between individuals on the same ward. Many staff reported good relationships with their immediate managers. The Rehabilitation ward emerged as maintaining a very supportive environment, with the staff support group also highly valued on this ward. On other wards comments were mixed, with some staff feeling under-valued:

*I think a major problem as well is that I think we're bending over backwards to look after the patients, but we're not being looked after. Breaks are really hard to take and it's just that more and more is being taken and it's a case of well, it's effective, and it's not really a case of 'you're doing a good job, so good on you'*. (Nursing Assistant, PICU)

#### The ward within the wider organisation

A view that senior managers, who were rarely seen on the wards, had a poor understanding of front-line work emerged on all seven wards:

*I just think sometimes the managers are up there, they have their job we have our job, but I don't think they understand what we really do. They'd have to spend like two weeks solid working with us 12 hours a day to understand what's going on*. (Staff nurse, Acute)

However, on the Rehabilitation ward, relative independence from senior management was also seen as having some advantages, with staff valuing their insularity and internal community.

Ward managers were also aware of the perceived remoteness of senior managers from front-line staff, feeling uncomfortably "sandwiched" between two tiers. They felt pressured from above by budgetary constraints and sometimes having to implement unpopular policies.

#### Having a Voice

Ward managers and other senior ward staff on every ward saw considerable benefits to morale of involving front-line staff in decision making, and described efforts to increase their currently limited 'voice' in the workplace:

*I think if the staff are not feeling contained and heard and as though they have a sense of agency, then it's almost as though they then can't give that to the patients that they're caring for and the whole thing falls apart - and I think, at times, it has felt very much like that*. (Clinical Psychologist, Acute)

One nursing assistant was frustrated at being excluded from ward rounds despite spending a great deal of time with patients:

*...I feel like I'm just here to go through processes and the mechanics of the day... I don't feel that I have an opinion that's really valued, or taken into account*. (Nursing assistant, Acute)

In general, for front-line staff, feeling unheard was more of an issue in relation to ward policies and organisation, especially workload, than clinical decisions.

*I would just like whatever issues I raise to be dealt with without me having to chase them up three or four times and it's really, like... You know, it kind of undermines... You feel, like, you know, no one cares*. (Nursing Assistant, Acute)

While negative comments predominated, some positive experiences were also reported. One ward has a system for lodging complaints or proposals to senior managers:

*We've got a good formal system management group with people high up in the Trust. So if you have a proposal it will be heard and taken seriously by management meetings. If they can't deliver at that meeting then it's certainly put on as an agenda item for another time*. (Social Worker, Child and Adolescent)

Experiences of feeling heard on other wards tended to be attributed to the approachability of particular senior ward staff.

### (c) Organisational structures

A further group of themes related to the definition of roles within ward teams and the protocols and guidelines in place for organising work on the ward.

#### Role Clarity and Confidence

Role clarity was highly valued throughout the sample, though only a minority currently described a lack of this. Managers were especially concerned with coupling responsibility with role clarity, and described strategies such as delegation of clinical and domestic responsibilities and the use of visual aids such as notice boards:

*And, I think, that's the thing for me, as well: give people the responsibility. But, in order to do that you have to explain to them what the responsibility entails. Don't just expect them to do something because if they don't understand why they're doing it and what the benefit is, and all that, they'll never really put their heart into it*. (Acute Care Service Manager)

As a caveat, staff did vary in the extent to which they *wanted *greater responsibility. One nursing assistant described her contentment with her role facilitating cooking groups:

*I'm quite happy with my job, being a nursing assistant and I even got a chance to go and do my training, I said I didn't want to. I'm happy with this job*. (Nursing Assistant, Forensic)

#### Consistency of structures

Consistent protocols and guidelines for organising work on the ward were found to help maintain clarity and confidence, whilst change was felt to create anxiety:

*If you can have cohesion in terms of a cohesive, communicating staff group and cohesion in the sense of structure, in terms of the way ward rounds (and) business meetings operate, that acts as a defence against the anxiety and chaos of psychosis. In my experience, that really assists the efficiency of the ward and that leads to pepping up and, sustaining morale*. (Consultant Psychiatrist, Acute)

Formal frameworks were also seen as vital for the maintenance of regular supervision and team meetings, which otherwise tended to fall by the wayside. Flexibility within a well-organised system was also valued, particularly in relation to shift systems. Several staff complained about a lack of flexibility around shifts, whilst a more flexible system on the Rehabilitation ward was seen as contributing to high morale.

On all seven wards there was discussion of recent structural or organisational changes and managers were aware that the frequent waves of change experienced by NHS staff, driven sometimes by central policy and sometimes by local reorganisation, have a considerable impact on staff.

#### Training

Opportunities for training were valued, and those in high-morale wards tended to be more positive about them. Ward managers also saw training positively as a way of improving standards, maintaining role clarity, imbuing confidence and maintaining morale. Good provision of mandatory courses was reported, but resource limitations restricted access to other courses, especially longer term ones, to which staff often had to dedicate their own time.

### (d) Being with patients

A final group of themes related to staff experiences of direct contact with patients on the ward.

#### Client Groups

The impact on staff of patients' severe disturbance was especially felt on acute and PICU wards:

*I think psychosis has a way of inducing chaos and fragmentation, and it's kind of like a manifestation of the condition but also, somehow that gets projected into structures and organisations and systems, in my experience, and there's plenty of room for chaos in a ward environment - especially within a busy ward environment*. (Consultant Psychiatrist, Acute)

Staff who had worked in a variety of settings commented that this made acute work more stressful, but some also valued the intensity of the work and pace of change:

*I've always loved this ward and the challenge, the busy-ness of the ward, you know, the range of people here*. (Charge Nurse, Acute)

#### Aggression and violence

The volatility of acute wards made violence frequent and risk highly salient to staff. A common sentiment running was that in cases of assault, "*there's no reparation really that can be made" *(Ward Manager, Child & Adolescent). Staff described how one or two individuals could shift the whole atmosphere of the ward:

*The worst time we had here was some time last year when we had a sort of gang mentality on the ward - like them and us, and that was pretty frightening sometimes really*. (Staff nurse, PICU)

Staff, especially on acute and PICU wards, appeared stoically to accept that some potential for violence was a given, but strategies for reducing risk were widely seen as inadequate. Higher staffing levels were seen as key, and some concerns were also raised about aspects of the physical environment, including locks and alarms:

*We haven't got enough staff, we haven't got enough time and we haven't got enough pagers and alarms to do it safely. That's the trouble*. (Staff nurse, Acute)

Knowing one's colleagues and feeling able to rely on them both for help in managing difficult situations and for emotional support was crucial. One nurse described how adverse incidents, when managed effectively, had the potential to enhance team morale:

*No incident is nice, but if we deal with it correctly and no one gets off really hurt or whatever and all the procedures are done, it's a good feeling. I think it's good because that shows we've got team work*. (Staff Nurse, PICU)

#### Dealing with social problems

Attitudes varied as to whether dealing with social problems was a legitimate role for ward staff. On the Rehabilitation ward, it was seen as rewarding:

*Here it's seeing people moving on and getting their own independence and living in their own flats and being a part of that really*. (Staff nurse, Rehabilitation)

On one acute ward however, suspicions were raised that some patients' problems were not really as they seemed: some service users were seen as 'using the system' to gain access to social resources, possibly preventing 'genuine patients' from accessing a bed.

#### Conversation and activities

Spending time with patients was seen as a core source of satisfaction; on six wards, staff felt that inadequate staffing and excessive administrative duties were impediments:

*You can't spend enough time with them and you're stressed out, and then that makes it even more stressful, because they're telling you you're basically not doing your job properly, because you're not spending time with us as much as you should*. (Healthcare assistant, Child & Adolescent)

On Rehabilitation and Child & Adolescent wards, staff valued having more time for this and for engaging in social and recreational activities with patients, building relationships with them. Staff who could find time for patients seemed to value their roles more and to see them as better defined.

*I was doing the cooking... and I remember one of the patients said, 'Today I felt like I'm a human being'. I said, 'Why are you saying that?' She said, 'You know the food that you gave me, it made me feel good, like I'm still alive.' *(Healthcare Assistant, Forensic)

#### Helping patients recover

Across all the wards, seeing patients get better was a positive influence on morale. Those working in Rehabilitation and Child & Adolescent units gained fulfillment from a long-term emotional investment in clients. For those in acute/intensive care, success was rated on a more short-term basis in terms of "stabilising" patients and discharging them home. Staff on these wards who maintained more consistent positive morale embraced the "challenge" of acute psychiatric care. Acute care staff were also more likely to see patients return to the ward. For a few, particularly on one acute ward, the "revolving door" phenomenon was a cause of frustration. Some felt disillusioned at the way factors beyond their control contributed to repeated readmission.

### (5) Physical Environment

Participants were also asked directly about the impact of the physical environment on their morale. Qualitative data pertaining to this topic will be reported elsewhere, so only a brief summary is given here.

A comfortable and attractive environment was not surprisingly seen as conducive to a good atmosphere, especially where staff and patients had access to outdoor space. Problems identified varied from ward to ward and included insufficient staff areas, poor air quality and lighting and lack of designated spaces for group activities or one-to-one sessions with patients. Particularly demoralizing were enduring problems, which could lead staff to feel neglected, though several people also described the joining effect of having to make do in adverse circumstances. Improvements to the physical environment were viewed as highly morale enhancing, sending a message that staff were valued.

## Discussion

### Main findings

The themes identified here both corroborate and extend previous findings on the factors affecting staff morale [10,17,18], and reassure us that some of the factors emerging as associated with morale indicators in our quantitative survey [13] are indeed likely to be causally linked to them. For front-line staff, the strongest positive influence on morale was peer support within a close knit team. Accounts sometimes brought to mind a fighting unit in combat - staff felt embattled and often neglected by senior leadership, but nonetheless maintained their morale through camaraderie, mutual loyalty and collaboration. On the whole, informal support from colleagues or managers, rather than formal supervision, provided the most comfort. Relationships with colleagues could transform the way difficult situations were experienced.

Participants emphasised the importance of role clarity and the structural and organisational factors needed to maintain it. Empowering staff and giving them greater autonomy was recognised as a way of enhancing morale, but only if responsibilities are clearly defined. These findings are congruent with and amplify those of our quantitative survey, where role clarity and autonomy, alongside strong team communication and support from colleagues, were closely associated with morale. Availability of supervision was not found to be associated with morale in our quantitative study. The qualitative findings suggest that supervision and training *can *be experienced as highly supportive in the context of the right working relationship and organisational context, but that front-line staff experiences of formal support mechanisms vary widely between individuals and between wards.

As in the quantitative study, our findings suggest that morale is influenced by a combination of individual job characteristics (limited resources available to meet the needs of demanding clients, mitigated by autonomy in a satisfying role and support from managers and above all colleagues) and broader organisational and cultural factors. This is congruent with recent research showing that factors such as workplace norms, role clarity, staffing resources, team communication and training opportunities have independent predictive value beyond the job-related variables of the Karasek model [21-23].

The emphasis placed on staffing in this qualitative study is at odds with the absence of any clear association between staffing and morale in our quantitative findings [14]. Reasons for this discrepancy are unclear, and it is possible that the lack of association in our quantitative findings may result from inadequate adjustment for factors producing variations in demands on staff between wards. Qualitative methods may better capture the wider effects of perceived staffing deficiencies on team morale and the working atmosphere. The feeling of being neglected or under-valued emerged as a particularly detrimental effect of perceived shortages and may be only partially reflected in standard quantitative measures of work stress.

Whereas contacts with colleagues were for the most part described very positively, experiences with patients were more diverse. Staff on acute and PICU wards spoke about the challenges posed by working with severely unwell clients, including the risk of violence. A stoical acceptance seemed to characterise many staff's attitudes towards this risk, but accounts suggested considerable limitations both in precautions against violence and in response to it. Lack of staff-patient contact time was a common complaint. Across all wards staff spoke about the rewarding nature of client contact and being motivated by helping patients recover.

### Limitations and strengths

The study's strengths are in the breadth of its sample, encompassing all levels of seniority and professions and several ward types. There is little previous relevant qualitative work and none of this scope. Constraints of space and the simple thematic nature of the analysis are limitations: we have had to focus only on the most prominent of the themes emerging from a large and rich data set. Selection of wards within the participating mental health Trusts was purposive, but considerations of convenience and feasibility also played a part in identifying the participating Trusts for this qualitative study.

### Implications for services

Ward team cohesion and mutual support and trust appear crucial to staff's ability to sustain and gain satisfaction from their roles in this potentially stressful environment. Much support seems to be informal, but trying to limit change in ward team composition or ensuring time and structures are in place for effective communication may reinforce it. Some hazards of such close-knit teams need also to be considered: high rates of bullying were reported in our quantitative study and staff who for any reason are perceived as outsiders by a very cohesive main group may be at risk from this.

As well as providing clear, sympathetic and flexible leadership, contributions that can be made by ward managers to staff well-being include implementing well organised ward routines and procedures, and attending to the clarity of staff roles and their ability to exercise some autonomy in carrying them out. Initiatives such as the recent Productive Ward initiative http://www.institute.nhs.uk/quality_and_value/productivity_series/productive_ward.html, aimed at redesigning processes across wards of a range of types, may support managers in this. The fit between a person's ideal conceptualisation of their role and the reality of that role in practice is important. Cahill et al [10] identified this as a recurring theme of qualitative studies carried out in different in-patient settings. Limited resources may force staff to compromise their ideal and changing structures and unreliable supervision may undermine their confidence in pursuing it. Where staff did not identify with and take ownership of their roles, they were likely to become demoralised. It was notable in those who felt positively about work that they had *chosen *to be where they were.

Staff's strong identification with the ward teams and their lack of a sense of 'voice' in the wider organisation may make new initiatives aimed at improving care difficult to implement, especially where they are seen as imposed by distant senior managers. Trade unions and professional bodies are a traditional conduit for worker voice, but no mentions were recorded of these in the qualitative study, suggesting they may not be prominent in the everyday life of the current NHS. Innovations for improving voice might include greater presence of senior managers on wards (the sense that they did not know what the life of the ward was like in practice was very strong among staff), opportunities for staff to be present at higher level Trust meetings, speak-up mechanisms allowing staff to get concerns and ideas for improvement heard by managers, more extensive consultations on important decisions and greater attention to the unions [24-26]. The position of ward managers in the organisation needs particular attention: their relationships with the rest of the ward team were often close, but their situation as 'middle managers' is a delicate and potentially stressful one to navigate.

Staff were not as satisfied with their relationships with patients as with colleagues, feeling especially that they lacked time to spend with them. This suggests that initiatives such as the Productive Wards programme (see above) and the recently promoted "Protected Engagement Time" (PET) scheme, aimed at ring-fencing time for patient contact [27] may have potential to improve morale as well as patient experiences. For these to be effectively implemented, it is important that staff feel able to dedicate time to individual patients without jeopardizing the safety of the ward. Regarding adverse aspects of patient contact, the staff narratives in our study followed much other evidence in suggesting that more needs to be done to reduce violence towards staff. Proposed avenues include closer links with police and more use of judiciously targeted prosecutions, security staff on wards, training for staff in reducing violence, environmental audits, greater attention to procedures for ensuring staff and patient safety, and clinical interventions targeting violence in specific clinical groups, such as patients with personality disorders [28-31]. Strategies also appear to be needed to improve the current rather passive and fatalistic response to staff who have experienced violence, for example by at least offering them supportive meetings with supervisors and/or managers and monitoring their response to incidents.

## Conclusions

Inpatient staff feel sustained in their potentially stressful roles above all by mutual loyalty and trust within cohesive ward teams. Clear roles, opportunities to work effectively with patients to achieve well-defined goals, supportive ward managers and well designed organisational procedures and structures also maintain good morale. Formal support mechanisms such as supervision and support groups are perceived as useful only to a limited extent by frontline staff. Perceived threats to good morale include staffing levels that are insufficient for staff to feel safe and able to spend time with patients, the high risk of violence, and lack of voice in the wider organisation.

Potential strategies for improving morale include increasing employee voice, designing jobs so as to maximise autonomy within clear and well-structured operational protocols, promoting greater staff-patient contact and improving responses to violence. These may have more to contribute to staff morale than a focus on formal support mechanisms. Intervention studies exploring ways of improving morale and links between staff morale and patient experiences and outcomes would be valuable.

## Competing interests

The authors declare that they have no competing interests.

## Authors' contributions

JT contributed to data collection and then led on analysing the data and drafting the paper, GLH was qualitative methods lead for the study, commenting on draft instruments and methods at all stages, contributing to the analysis and commenting on drafts of this paper, EW contributed to development of methods and instruments for the paper, collected the majority of the data and commented on drafts of the paper. MP contributed to the development of the protocol and study instruments, supervised the researcher collecting data at three sites, contributed to the analysis and commented on drafts. SJ was Principal Investigator for the study and led on development of the original protocol, and she then contributed to methods and instruments, supervised EW and JT, contributed to data analysis and helped draft the paper. All authors have approved the final draft.

## Endnotes

^1 ^Standardised mean morale scores were calculated by standardising each morale indicator so that scores were distributed on a 1 to 100 scale, reversing direction where appropriate so that a higher score indicated better morale, and then calculating for each ward the mean score for all the morale indicators. The morale indicators used in this calculation included measures of dimensions of burnout, intrinsic job satisfaction, work-related well-being, job involvement and general emotional well-being: details and references are given in the study final report [13].

## Pre-publication history

The pre-publication history for this paper can be accessed here:

http://www.biomedcentral.com/1471-244X/11/68/prepub
